# Past, Present, and Future of Familial Hypercholesterolemia Management

**DOI:** 10.14797/mdcvj.887

**Published:** 2021-09-24

**Authors:** Viviane Z. Rocha, Raul D. Santos

**Affiliations:** 1 Heart Institute (InCor) University of Sao Paulo Medical School Hospital, Sao Paulo, Brazil; 2 Hospital Israelita Albert Einstein, Sao Paulo, Brazil

**Keywords:** cholesterol, atherosclerosis, genetics, calcium score, statins, PCSK9

## Abstract

Familial hypercholesterolemia (FH) is a monogenic form of severe hypercholesterolemia that, if left untreated, is associated with early onset of atherosclerosis. FH derives from genetic variants that lead to inefficient hepatic clearance of low-density lipoprotein (LDL) particles from the circulation. The FH phenotype is encountered in approximately 1 of every 300 people. The risk of atherosclerotic cardiovascular disease (ASCVD) is higher in those with FH than in normolipidemic individuals and in those with polygenic hypercholesterolemia. FH is usually diagnosed by clinical scores that consider hypercholesterolemia, family history of early ASCVD and hypercholesterolemia, and cutaneous stigmata. Genetic diagnosis is important and should be offered to individuals suspected of FH. Family cascade screening is important to identify asymptomatic hypercholesterolemic individuals. Despite the high risk of ASCVD, this risk is heterogenous in heterozygous FH and depends not only on high LDL cholesterol (LDL-C) but also on other risk biomarkers. Risk can be evaluated by considering biomarkers such as male sex, late-onset therapy (> age 40), LDL-C > 310 mg/dL, low high-density lipoprotein cholesterol, elevated lipoprotein(a), obesity, diabetes, and hypertension by using specific risk equations and by detecting subclinical coronary atherosclerosis. Statins are the main therapy for FH and change the natural history of ASCVD; however, most individuals persist with elevated LDL-C. PCSK9 inhibitors provide robust and safe LDL-C lowering in FH, although elevated costs preclude their widespread use. Newer therapies such as ANGPTL3 inhibitors add intensive LDL-C lowering for refractory forms of FH. Finally, while it is possible to normalize LDL-C in people with FH, the disease unfortunately is still severely underdiagnosed and undertreated.

## Introduction

Familial hypercholesterolemia (FH) is classically defined as a monogenic form of severe hypercholesterolemia and is characterized by the presence of very high levels of plasma low-density lipoprotein cholesterol (LDL-C).^1^ Because FH is an inherited form of hypercholesterolemia, where the majority of affected individuals have hypercholesterolemia at birth, they sustain a long exposure to high LDL-C levels that, if left untreated, dramatically increases the risk of premature atherosclerosis and its consequences. Early diagnosis and proper treatment may suffice to attenuate and often abrogate the excess risk associated with this genetic condition.^2,3^

FH derives from genetic variants that lead to inefficient hepatic clearance of LDL particles from circulation. Autosomal dominant variants in the LDL receptor gene (*LDLR*) represent the most common cause of FH and for decades were recognized as a single etiology of monogenic FH.^1^ Later, identification of causative variants in the apolipoprotein B gene (*APOB*), and more recently in the proprotein convertase subtilisin kexin 9 gene (*PCSK9*), expanded the list of FH causal genes.

FH is highly prevalent, with estimated frequency of approximately 1 affected individual in every ~300 people from the general population.^4,5^ Nevertheless, most epidemiologic studies examine FH frequency based solely on phenotype, without considering molecular genetic criteria.^6^ Since a substantial proportion of patients with possible or probable heterozygous FH by clinical criteria actually have no detectable monogenic cause, the precise estimation of FH prevalence depends on the definition used to diagnose this condition, whether clinical, genetic, or a combination of both. Whereas a clinical diagnosis for FH is critical for practical reasons, the fact that the majority of these individuals should receive lipid-lowering therapies means that identifying genetically confirmed FH is a step further in recognizing and treating individuals with a higher risk of atherosclerotic cardiovascular disease (ASCVD).^7^ A genetic diagnosis also has implications for the success of cascade screening in possibly affected relatives.^8^ The availability of modern potent lipid-lowering therapies, particularly PCSK9 inhibitors,^9^ in addition to recognizing the impact of monogenic defects and the heterogeneity of ASCVD risk in FH,^10^ have expanded the utility of identifying higher-risk individuals beyond the prognostic dimension, paving the road towards more personalized medicine.

## Pathophysiology

Biologically, LDL particles consist in a primordial element in the atherosclerotic process, penetrating and accumulating in the arterial intima, where oxidation and other biochemical modifications can render them proinflammatory and susceptible to phagocytosis by local macrophages.^11^ Accumulation of these lipid-overloaded cells, the so-called foam cells, in the subendothelial space is the first sign of an atherosclerotic plaque. In addition to the biological plausibility, multiple lines of evidence have associated plasma LDL-C levels with ASCVD; more recently, however, Mendelian randomization studies have further dissected the importance of cumulative exposure to hypercholesterolemia.^12^ In FH, high LDL-C and long exposure is determinant of increased ASCVD risk.

The molecular basis of increased levels of LDL-C in FH usually derives from genetic variants in the *LDLR*, leading to a reduced number or activity of LDL receptors and ultimately culminating in diminished hepatic clearance of LDL particles from the blood.^1^ Other established molecular mechanisms resulting from variants in the *APOB* or gain-of-function variants in *PCSK*9 also lead to hypercholesterolemia due to impaired hepatic uptake of circulating LDL. Causal variants in all three genes are considered codominant, meaning that in the case of two mutated alleles (one from each parent), both contribute to the magnitude of phenotype.^13^

### Causal *Genes*

#### LDLR

Approximately 60% to 80% of subjects with heterozygous FH present variants in the *LDLR* gene. A long list of over 2,000 rare variants in *LDLR* have already been reported, including missense variants as the most frequent variant type, but also nonsense variants, splicing variants, small insertions or deletions, and large-scale DNA copy number variations (CNVs).^13^ The pathogenic variants in *LDLR* can impair any step of LDLR-mediated endocytosis of LDL particles. Overall, variants can be functionally categorized in two groups: receptor-negative or null variants (characterized by no synthesis or synthesis of a nonfunctional receptor) and receptor-defective variants (characterized by synthesis of a defective, though still functional, receptor). In general, carriers of receptor-negative variants present the highest levels of LDL-C.

#### *APOB, PCSK9*, and Other Genes

Variants in *APOB* are present in approximately 5% to 10% of individuals with a clinical diagnosis of heterozygous FH.^1^ The *APOB* variants lead to a defective apolipoprotein B100, a crucial ligand for LDLR, and often induce a milder phenotype when compared to *LDLR* variants. PCSK9 gain-of-function variants represent a rare cause of FH, contributing to less than 1% of FH cases.

*LDLRAP1*, which encodes LDL receptor adaptor protein 1, is another gene that very rarely can be implicated in FH.^13^ Variants in *LDLRAP1* lead to an autosomal recessive form, also called ARH, clinically characterized by severe hypercholesterolemia and genetically recognized as a homozygous form of FH.

*ABCG5, ABCG8*, and *LIPA* genes are also implicated in phenotypes that are similar to FH and therefore called phenocopies but are characterized by a recessive inheritance. Variants in *ABCG5* and *ABCG8* can cause sitosterolemia, a rare disease characterized by increased circulating levels of plant sterols,^14^ and variants in *LIPA* can induce a disease characterized by cholesterol ester storage due to lysosomal acid lipase deficiency.^15^ Finally, rare variants in *APOE* have also been associated with FH-like phenotypes.^13^ Of interest, the *STAP1* gene recently suffered a downgrade as a possible cause of the FH phenotype since no cosegregation of high LDL-C was encountered in individuals bearing genetic variants within the same family or in experimental animals.^16,17^

#### Polygenic Hypercholesterolemia

Polygenic hypercholesterolemia generally derives from the presence of multiple, small-effect, and common single-nucleotide polymorphisms (SNP) instead of single, large-effect, and rare variants that characterize monogenic hypercholesterolemia.^8^ Although generally associated with a less-severe phenotype, a high number of SNPs in polygenic hypercholesterolemia can also lead to LDL-C levels in the magnitude observed in the monogenic forms.^6^ However, transmission within the family is not so straightforward as in monogenic defects,^6,8^ and ASCVD risk seems to be lower than the one in FH despite elevated LDL-C concentrations.^7,18^

## Epidemiology

Among the rare genetic disorders, FH is probably the most common. Early studies approximated the prevalence of FH at ~ 1:500 in the general population, but this estimate was derived mostly from limited data.^6^ In 2020, two large meta-analyses comprising millions of individuals suggested worldwide estimates of FH prevalence in the general population and in subjects with ASCVD.^4,5^ Despite mild differences in the number of included studies and comprised individuals, both meta-analyses estimated a pooled FH prevalence of 0.32% (corresponding to 1:313 in the study by Beheshti et al. and 1:311 in the study by Hu et al.). Heterogeneity was high across the included studies, likely resulting from multiple differences between the diagnostic criteria used in each study as well as study populations and designs, among others. Since the risk of atherosclerotic events is significantly enhanced in the FH population, it is plausible to hypothesize a higher prevalence of FH among those with established ASCVD, a finding corroborated by both studies. In the study by Beheshti et al.,^5^ the prevalence of FH was 1:31 in those with ischemic heart disease (10-fold higher than the general population), 1:15 in those with premature ischemic heart disease (20-fold higher than the general population), and 1:14 in those with severe hypercholesterolemia. In the study by Hu et al.,^4^ the pooled FH prevalence among those with ASCVD was 1:17, 18-fold higher than the general population. Despite the large number of studies and subjects in these contemporary meta-analyses, data from most other countries are not available, and thus FH prevalence is still unknown in several regions of the world.

## Diagnosis

### Clinical Scores

Clinical scoring systems to diagnosis FH are widely used and accepted, and multiple sets of criteria including clinical, biochemical, and genetic parameters have been developed.^1,19^ In addition, a personal history of early ASCVD, a family history of premature ASCVD and/or severe hypercholesterolemia, and the presence of physical stigmata (xanthomas or corneal arcus) represent important FH diagnostic criteria along with persistent levels of high LDL-C. Considering the practical difficulties in assessing some of these criteria, simpler criteria were proposed^19^: heterozygous FH may be diagnosed in a child in the presence of a positive family history of elevated cholesterol or premature coronary artery disease and LDL-C ≥ 160 mg/dL or in an adult with the same above criteria and LDL-C ≥ 190 mg/dL (confirmed on two occasions). Nonetheless, ruling out the possibility of a secondary cause of severe hypercholesterolemia, such as hypothyroidism, nephrotic syndrome and cholestatic hepatic disease, is a *sine qua non* before considering the diagnosis of FH.

Despite subtle diagnostic differences between the most-used FH scores, the Simon Broome Register (SBR) and the Dutch Lipid Clinic Network (DLCN) criteria also take into account the presence of *LDLR, APOB* and *PCSK9* variants in their scoring systems.^13^

### Genetic Testing

The identification of an FH-causing variant in suspected cases is highly variable depending on the nature of the cohort. In a large-scale sequencing study by Khera et al., only approximately 2% of individuals with severe hypercholesterolemia from the general population, defined as untreated LDL-C ≥ 190 mg/dL, showed a pathogenic variant in an autosomal dominant FH gene.^7^ Conversely, in another study using next-generation sequencing on referred patients with severe hypercholesterolemia, Wang et al. found a monogenic FH-causing variant in 47.3%; this percentage increased to 53.7% when analysis of copy number variations were included.^20^

Classic FH presents as severe hypercholesterolemia resulting from a pathogenic variant in one of the FH-implicated genes. Even so, the role of DNA sequencing in the diagnosis of FH is debated.^1^ While genetic testing is recommended by some experts whenever FH is suspected,^21^ and several countries consider identification of an FH-causing variant to be the gold standard in FH diagnosis, there is still no consensus on its utility. The low use of genetic testing in clinical practice has been attributed to its limited availability, elevated cost, and uncertainty about variant pathogenicity.^22^ Furthermore, theoretical concerns about possible discrimination in obtaining medical and life insurance for someone with a “genetic disease” may negatively influence one’s pursuit of genetic testing.^23^

Nevertheless, the association between the presence of a pathogenic variant in one of the implicated FH genes and a higher ASCVD risk is undeniable,^7,18^ which may be reason enough to consider genetic testing whenever it is available and affordable. In the study by Khera et al., subjects with LDL-C ≥ 190 mg/dL and no variant had a 6-fold higher CAD risk relative to those with LDL-C < 130 mg/dL (OR 6.0; 95% CI, 5.2-6.9), whereas participants with LDL-C ≥ 190 mg/dL associated with an FH variant presented a 22-fold higher CAD risk (OR 22.3; 95% CI, 10.7-53.2) compared to the same reference group.^7^ Additionally, Trinder et al. evaluated the impact of monogenic and polygenic causes of hypercholesterolemia on premature ASCVD events in individuals with clinically diagnosed FH.^18^ While a monogenic cause of FH was associated with significantly higher risk of cardiovascular disease (adjusted hazard ratio: 1.96; 95% CI, 1.24-3.12; *P* = .004), those patients with polygenic hypercholesterolemia did not have a significantly different risk from those with no identified genetic cause of FH. However, patients with both monogenic FH and a high polygenic score were at highest risk of early cardiovascular disease.

### Cascade Screening

Given the autosomal-dominant pattern of FH, once an index case is detected, all first-degree relatives should undergo cascade screening to identify those who may be affected. Cascade screening has been shown to be a cost-effective manner to identify asymptomatic family members with FH. This can be done by either cholesterol screening or genetic testing.^1^ The latter is important since it (1) facilitates a definitive diagnosis mainly in pubertal children and adolescents, (2) identifies higher-risk individuals rather than those with polygenic hypercholesterolemia or those in whom no molecular defects are found, (3) may increase initiation and adherence to therapy, and (4) increases the efficacy of the cascade screening process.^6,21^ One important yet debatable issue is that of universal cholesterol screening in childhood, which is recommended by the National Heart, Lung, and Blood Institute; the National Lipid Association Expert Panel; and the European Expert Panel.^24^ The high cholesterol discrimination in children with and without FH and lower frequency of confounders makes cholesterol screening an ideal opportunity for early diagnosis, as does reverse cascade screening, which is effective in detecting FH in first- and second-degree relatives of children identified with FH and may improve case findings as shown in the study by Wald et al. in the UK.^25^

## Natural History and Heterogeneity of ASCVD Risk in FH

Classically, FH is associated with a high risk of early onset ASCVD.^2^ When defined by clinical criteria, untreated FH is associated with a 3.2-fold greater risk of coronary heart disease mortality compared with the general population^26^ in the UK. The risk is proportionally greater in younger individuals with FH (ie, 5 vs 0.03 expected deaths, 12.35 standardized mortality rate for those aged 20 to 39 years [95% CI, 5.14-29.70]) compared with non-FH populations. This risk decreased to 2.17 (95% CI, 1.17-4.03), or 10 versus 0.4 expected deaths, and 1.19 (95% CI, 0.53-2.65), or 6 versus 4.62 expected deaths, for those aged 40 to 59 and 60 to 69 years, respectively, as shown in Norway over a 20-year period.^27^

Except for the rare homozygous FH phenotype—where the risk of early ASCVD is almost universal^10,28,29^ and highly dependent on LDL-C concentrations—the risk in heterozygous FH is heterogeneous and depends on many factors.^10,30,31,32,33^ Prospective studies have shown that even for individuals with confirmed genetic defects, this risk depends not only on higher LDL-C but also on male sex,^30,33^ late treatment onset,^10^ smoking,^30,32^ higher lipoprotein(a) levels,^30,34^ presence of type 2 diabetes or hypertension,^26^ lower HDL-cholesterol,^35^ and the presence of subclinical coronary atherosclerosis.^31^

### Risk Stratification in FH

It is universally accepted that those with a diagnosis of heterozygous FH must be treated with statins or ezetimibe to reduce LDL-C by at least 50%.^10,36^ Indeed, observational data from the Netherlands suggest that this reduces the risk of myocardial infarction to the level of the general population.^3^ Despite this, many individuals with FH persist with an elevated risk of ASCVD and mortality.^10^ This is especially true for those with previous ASCVD manifestation,^30^ very high LDL-C,^10^ and the presence of risk biomarkers such as coronary artery calcification (CAC).^31^

There are many risk equations to help diagnose FH in various geographic regions. Risk equations specific to FH, such as the SAFEHEART-Risk Equation, have been validated for Spanish^30^ and French^37^ populations. The MONTREAL FH score was validated in Canada.^35^ The Severe FH definition from the International Atherosclerosis Society^10^ has been shown to identify higher-risk FH populations in the UK’s Simon Broome cohort,^38^ whereas the CAC score has been shown to identify higher-risk FH individuals in Brazil, France, and Spain.^31,39^ Indeed, a CAC score of zero, which occurs in approximately 45% of individuals enrolled in published imaging FH cohorts,^40^ has been associated with a very low risk of ASCVD events in those undergoing standard LDL-C–lowering therapy despite having elevated LDL with treatment after a median 2.7 to 3.7-year follow-up. ***Table 1*** shows different biomarkers to identify individuals with FH who are at higher cardiovascular risk.

**Table 1:** Biomarkers to identify individuals with familial hypercholesterolemia who are at higher risk of major adverse cardiovascular outcomes. CAC: coronary artery calcification; LDL-C: low-density lipoprotein cholesterol; HDL-C: high-density lipoprotein cholesterol; ASCVD: atherosclerotic coronary vascular disease; Lp: lipoprotein; IAS: International Atherosclerosis Society; MESA: Multiethnic Study of Atherosclerosis.


LDL-C AND TREATMENT ONSET^10^	RISK BIOMARKERS^32,41^	RISK EQUATIONS^30,35^	IAS SEVERE FH DEFINITION^10^	CAC SCORES^31,39,43^

Usually ≥ 310 mg/dL Late onset of LDL lowering (> 40 years old)	Male sex Older age Smoking Hypertension Diabetes High Lp(a) Low HDL-C	SAFEHEART risk equation^30^ Previous ASCVD On treatment LDL-C (≥ 160 mg/dL) Higher body mass index Lp(a) > 50 mg/dL Hypertension Smoking MONTREAL FH Score^35^ Age Male sex Low HDL-C Hypertension Smoking	Previous ASCVD defined as myocardial infarction, angina, coronary revascularization, nonembolic ischemic stroke, or transitory ischemic attack, and intermittent claudication Advanced subclinical atherosclerosis (CAC > 100 or > 75th percentile for age and sex according to MESA, or computer tomography angiography with obstructions > 50%, presence of nonobstructive plaques in more than one vessel) LDL-C > 400 mg/dL, or LDL-C > 310 mg/dL and one high-risk feature,* or LDL-C > 190 mg/dL and two high-risk features*	CAC > 0 CAC > 100


* Risk features: age > 40 years without treatment, smoking, male sex, lipoprotein(a) > 125 nmol/L (50 mg/dL), HDL-C < 40 mg/dL), hypertension, diabetes mellitus, family history of early cardiovascular disease in first-degree relatives (age < 55 years in men and < 60 years in women), chronic kidney disease (ie, estimated glomerular filtration rate < 60 mL/min per 1·73 m^2^, and BMI > 30 kg/m^2^

### Current and Future Therapies

Despite the efficacy of statins and ezetimibe, most individuals with FH will not achieve proposed LDL-C goals for prevention of ASCVD events. In the SAFEHEART registry, roughly 5% and 22% of patients achieved LDL-C < 70 and < 100 mg/dL, respectively.^41^ Monoclonal PCSK9 inhibitors may add 50% to 55% additional LDL-C lowering for individuals with severe heterozygous and 22% for those with homozygous FH.^9^ In a recent evaluation from the same SAFEHEART cohort, the addition of PCSK9 inhibitors on top of usual therapy helped 67% of patients attain LDL-C < 70 mg/dL and 80% attain LDL-C < 100 mg/dL.^42^ In those considered to be at very high-risk, LDL-C < 55 mg/dL was attained in 46% of the cases.

Although monoclonal PCSK9 inhibitors are a robust and safe therapy for those with heterozygous FH, their high cost precludes widespread use. Therefore, to improve cost-effectiveness, we have proposed risk stratification in people with FH without previous ASCVD events using mainly CAC scores to identify those with higher risk.^43^ In this scenario, individuals with CAC > 0 who persist with inadequate LDL-C would be prescribed PCSK9 inhibitors, whereas use of PCSK9 inhibitors would be postponed in the absence of CAC testing and result in longer-term follow-up.

Inclisiran is a small interfering RNA (siRNA) molecule that blocks the hepatic production of PCSK9, thereby reducing LDL-C by 50% in patients with heterozygous FH.^44^ Its advantage compared with monoclonal inhibitors is its dosage, requiring only 2 to 3 injections per year instead of every 2 or 4 weeks with the latter. So far, no safety events have been described, and inclisiran is approved in Europe.

One of the greatest challenges is the treatment of homozygous FH, especially in patients carrying two null alleles on *LDLR*. These patients are refractory to PCSK9 inhibitors and usually need drugs to block very low-density lipoprotein synthesis, such as lomitapide, and LDL apheresis. Indeed, LDL apheresis is highly efficacious and should prolong life in those with severe forms of FH, although it must be performed weekly to avoid LDL-C rebound.^45^ Recently evinacumab, a monoclonal antibody against ANGPTL3 (angiopoietin-like protein 3), was shown to reduce LDL-C by 50% in refractory homozygous FH patients, including those with null *LDLR* variants.^46^ Recently approved for homozygous FH, evinacumab will be an enormous benefit for these patients as well as those with refractory heterozygotes.^47^ In the future, genetic editing therapies such as CRISPR/Cas9 or gene transfection may change the natural history of FH for good.^48^ At present, however, FH is still underdiagnosed and undertreated.^49^

## Conclusions

FH is a frequent autosomal genetic disorder that causes early ASCVD. It is important to differentiate it from other forms of severe hypercholesterolemia since it may have severe implications for both index cases and relatives. The risk of ASCVD in FH depends not only on high LDL-C levels but also on other biomarkers, especially in the presence of subclinical atherosclerosis. Statins have dramatically changed the natural history of FH, although for many individuals these drugs are not enough to mitigate ASCVD risk. The addition of PCSK9 inhibitors to statins and/or ezetimibe introduced a new era in which LDL-C finally can be controlled in heterozygous FH, and risk stratification may help improve the cost-effectiveness of newer lipid-lowering therapies.

## Key Points

- Familial hypercholesterolemia (FH) is characterized by high cholesterol since birth.
- FH causes early atherosclerosis and mortality.
- Monogenic hypercholesterolemia bears a greater cardiovascular risk than polygenic hypercholesterolemia.
- Atherosclerosis risk is heterogeneous in FH.
- High levels of low-density lipoprotein cholesterol must be aggressively treated in FH.
